# Comparison of the effects of autologous and allogeneic purified platelet-rich plasma on cartilage damage in a rabbit model of knee osteoarthritis

**DOI:** 10.3389/fsurg.2022.911468

**Published:** 2022-07-15

**Authors:** Lingling Wang, Luting Zhao, Lianwei Shen, Qilin Fang, Zhenglei Yang, Rongrong Wang, Qing Wu, Yulei Xie

**Affiliations:** ^1^ Department of Rehabilitation Medicine, Affiliated Hospital of North Sichuan Medical College, Nanchong, China; ^2^ Department of Rehabilitation Medicine, The First People’s Hospital of Ziyang, Ziyang, China; ^3^ Department of Rehabilitation Medicine, The Affiliated Hospital of Jinzhou Medical University, Jinzhou, China; ^4^ Department of Rehabilitation Medicine, Shandong University Cheeloo College of Medicine, Jinan, China; Department of Rehabilitation Medicine, Affiliated Hospital of North Sichuan Medical College, Nanchong, China; ^5^ Faculty of Rehabilitation Medicine, Capital Medical University, Beijing, China

**Keywords:** knee osteoarthritis, purified platelet-rich plasma, BMP-2, sox9 knee osteoarthritis, SOX9

## Abstract

**Background:**

Purified platelet-rich plasma (P-PRP) is gradually being used in the treatment of osteoarthritis (OA), and its sources are mainly divided into autologous and allogeneic blood. However, it is unclear whether autologous PRP is more effective or allogeneic PRP is superior.

**Objective:**

In this study, autologous and allogeneic P-PRP was injected at early stage of KOA in rabbits, and then the differences in the efficacy of the two P-PRPs against KOA were compared from several perspectives, including pathological histology and immunohistochemistry.

**Method:**

Experimental rabbits were divided into normal group (*n* = 8), model group (*n* = 8), autologous P-PRP group (*n* = 8), and allogeneic P-PRP group (*n* = 8) using a random number table method. The normal and model groups did not receive any treatment, and the autologous P-PRP and allogeneic P-PRP groups received intra-articular injections of autologous and allogeneic P-PRP, respectively, to observe the changes in the gross specimens of the knee joints of the experimental rabbits in each group. The histopathological changes of chondrocytes were also observed by HE-stained sections of articular cartilage, and the expression of chondrocytes Bone morphogenetic protein-2 (BMP-2) and Sox9 were detected by immunohistochemistry.

**Results:**

Compared with the allogeneic P-PRP group, the differences were statistically significant (*P* < 0.05) in the gross specimens and pathological histological findings in the autologous PRP group. Immunohistochemical results showed that the expression of BMP-2 and Sox9 was elevated in both the autologous P-PRP group and the allogeneic P-PRP group compared with the model group, and the expression of BMP-2 was higher in the autologous P-PRP group than in the allogeneic P-PRP group, with a statistically significant difference (*P* < 0.05), while there was no difference in the expression of Sox9 between the two groups (*P* > 0.05).

**Conclusion:**

Intra-articular injection of autologous P-PRP activated the expression of BMP-2 and Sox9 in chondrocytes and effectively improved KOA cartilage repair and reduced bone redundancy and joint fluid formation, and its efficacy was superior to that of intra-articular injection of allogeneic P-PRP.

## Introduction

Osteoarthritis (OA) is a common degenerative joint disease, with intra-articular cartilage damage as the main manifestation [1]. It includes changes in cartilage structure, gene expression patterns and epigenetic regulation [2]. Currently, more than 300 million people worldwide have OA [3]. With the increasing aging of society and the increasing number of obese people, the incidence and prevalence of OA continue to rise. However, there are still some limitations in the commonly used treatment methods [4]. Therefore, it is necessary to explore new approaches to treat OA.

Platelet-rich plasma (PRP) is obtained by multiple centrifugations of peripheral blood. Activated PRP can release a variety of growth factors, cytokines and related signaling molecules, making it useful in KOA therapy to promote cartilage repair and regulate the inflammatory response, etc [5–7]. PRP is divided into pure platelet-rich fibrin (P-PRF), leukocyte and platelet-rich fibrin (L-PRF), purified platelet-rich plasma (P-PRP), leukocyte and platelet-rich plasma (L-PRP), and in recent years P-PRP has been gradually applied to KOA [8–11]. Currently, most of the P-PRP used for clinical and research purposes are derived from autologous blood, but it is difficult to prepare autologous P-PRP for patients with blood disorders or frailty, so allogeneic PRP has come into being. However, the quality of allogeneic PRP varies due to its individual differences, which may affect the clinical efficacy of P-PRP.

The aim of this study is to compare the efficacy of autologous PRP and allogeneic PRP in the treatment of KOA, to initially explore whether allogeneic P-PRP can replace autologous P-PRP, and to provide some theoretical basis for the subsequent standardized preparation of allogeneic P-PRP.

## Methods

### PRP preparation

During the study, the preparation of PRP was operated in a room temperature 22°C environment, and all operations were completed in a biosafety cabinet, and 6 ml of the collected arterial blood was transferred to 15 ml sterile centrifuge tubes. The PRP was prepared by the secondary centrifugation method reported by Perez et al. [12]. 5–6 ml of arterial blood was collected from the proximal 1/3 of the central artery of the rabbit's ear as a puncture point, and the collected arterial blood was transferred to a 15 ml sterile centrifuge tube (containing 0.8 ml of 3.2% sodium citrate solution), and the first relatively low-speed centrifugation (100 × g, 1,050 r/min, 8 cm centrifugation radius) was performed. After discarding the erythrocyte layer a second relatively high speed centrifugation was performed (400 × g, 2,100 r/min, 8 cm centrifugal radius), both times for 10 min. The platelet-rich plasma layer was deposited at the bottom of the tube, and 0.5 ml of plasma was left to shake well with the platelet-rich plasma layer to obtain P-PRP. Prior to joint cavity injection. P-PRP was activated with 10% calcium chloride solution in a 9:1 ratio [13]. In this study, pre-experiments have been conducted in the previous stage, i.e. 5–6 ml of whole blood was collected from the central artery of rabbit ear, 0.5 ml of P-PRP was obtained according to the P-PRP preparation method, and the obtained P-PRP was subjected to whole blood cell analysis, and the results showed that the concentration of platelets in P-PRP was (1,987 ± 219.48) × 10^9^/L, the concentration of leukocytes was (0.14 ± 0.08) × 10^9^/L, the concentration red blood cell concentration was (0.1 ± 0.05) × 10^9^/L.

### Intra-articular cavity injection of PRP

The skin of the knee joint of the right hind limb of the experimental rabbits was prepared 1 day before treatment, and the skin around the knee joint was fully exposed and handled carefully to avoid damaging the skin and causing infection.

The experimental rabbits were fixed in the rabbit box with the right knee joint flexed at 40°–45°, and the inner edge of the patellar tendon ligament at the inferior pole of the patella was used as the entry point and marked. After routine disinfection and spreading a towel, take a 1 ml syringe and slowly enter the joint cavity from the puncture point, remove the syringe and replace it with a syringe containing activated P-PRP (activate 0.5 ml of P-PRP with approximately 0.056 ml of 10% calcium chloride solution and mix well to be ready for use). During the injection, the skin should be lifted close to the needle to prevent drug leakage. After injection, the puncture needle is removed, the puncture site is cauterized, the dressing is covered, and the rabbit knee joint is passively flexed and extended several times so that P-PRP spreads uniformly throughout the joint cavity and becomes a gel.

### Animals and experimental design

This study followed the National Institutes of Health Guide for the Care and Use of Laboratory Animals (NIH Publication No. 85–23, revised 1996) and the Chinese Ministry of Health Animal Management Regulations, and was approved by the Ethics Committee of Ziyang First People's Hospital. Thirty-four clean-grade New Zealand healthy large-eared rabbits, aged 3 months and weighing 2.48 ± 0.21 kg, were purchased from the Laboratory Animal Center of Southwest Medical University (Certificate of Conformity No. SCXK[Chuan] 2013–065). All rabbits were kept in single cages at a temperature of 22°C–26°C, with humidity maintained between 40%–60%, adequate feed, clean water, and excreta cleaned daily. During the experiment, every effort was made to limit the number of animals used and reduce suffering. Humane endpoints were set; animals were humanely euthanized when they suffered chronic or severe pain or distress during the experiment.

In this study, a rabbit KOA model was established by intra-articular injection of a mixture of papain and L-cysteine. The 5% papain, 0.03 mmol/L L-cysteine (ready-to-use), and the two were mixed in a 2:1 ratio, allowed to stand for 30 min, filtered through a 0.22 um filter membrane and then kept for use. The mixture of 5% papain and 0.03 mmol/L L-cysteine (0.1 ml/kg) was injected intra-articularly on days 1, 3 and 5, respectively, for a total of three times [14, 15].

After 34 healthy New Zealand rabbits were acclimatized and fed for one week, 8 of them were randomly selected as the normal control group using the random number table method, and the remaining 26 were established as KOA models. 2 weeks after the first papain injection, 2 experimental rabbits were randomly selected and executed to observe the right knee joint lesions in rabbits, and after successful modeling was determined, 24 rabbits were divided into model group (*n* = 8), autologous P-PRP group (*n* = 8), and allogeneic P-PRP group using the random number table method (*n* = 8), and allogeneic P-PRP group (*n* = 8). The normal group and the model group don't receive any treatment. In the autologous P-PRP group and allogeneic P-PRP group, they received 0.5 ml of autologous P-PRP and allogeneic P-PRP by injection into the knee joint cavity once every 2 weeks for 3 times.

### Gross specimen observation

Two weeks after the final intervention treatment, the experimental rabbits in each group were executed by air embolization, and the knee joint cavity of the rabbits was opened layer by layer using the medial femur as an incision, and the joint fluid and articular cartilage surface were observed visually and rated with reference to the OARSI macroscopic rating system for articular cartilage, bone cambium, and joint fluid [16].

### Femoral end cartilage pathology evaluation

The femur and tibia were dissociated at about 1 cm from the articular surface, the femoral ends were taken and placed in 10% formaldehyde solution for 24 h and then removed, the specimens were rinsed with running water for 1–2 h and then immersed in 10% EDTA decalcification solution for 2 months, the decalcification solution was changed regularly every week, and after complete decalcification (no resistance to large-headed needle puncture into the specimen), immersion wax embedding, sectioning, alcohol dehydration step by step, hematoxylin-eosin staining was performed, and pathological tissue sections were observed by implementing blind and double observation, and the observations were rated with reference to Mankin's scoring criteria [17].

### Immunohistochemical assay

Paraffin sections were dewaxed and dehydrated, and a series of antigen repair, blocking, dropwise addition of primary and secondary antibodies, color development, dehydration, blocking, and immunohistochemical detection were performed, and the distribution and changes of BMP-2 and Sox9 expression in each group of chondrocytes were observed using a light microscope under a 400× field of view, and Image Pro Plus 6.0 image Image Pro Plus 6.0 image analysis software was used to calculate the mean optical density values of BMP-2 and Sox9 in each group, and the mean values were calculated separately, and then taken as the final measurement value of a single slice; to avoid the influence of subjective factors, a blind method was applied to the evaluator during observation.

### Outcomes

The primary outcome was histopathology and immunohistochemistry. Secondary outcomes were gross specimen observations.

### Statistical analysis

Statistical analyses were performed using SPSS version 26 (IBM Corp, Armonk, NY, USA) for statistical analysis, and continuous variables were expressed as mean versus SD or median versus interquartile range. For comparison of measures between multiple groups, one-way analysis of variance (One-way-ANOVA) was used if normality and chi-square conditions were satisfied, and SNK-q test was used for comparison between two groups. If normality and homogeneity of variance were not satisfied, Kruskal-Wallis test was used for comparison between multiple groups and Mann-Whitney test for comparison between two groups; Kruskal-Wallis test was used for comparison between multiple groups and Mann-Whitney test for comparison between two groups for rank data. *P* < 0.05 was considered statistically significant. *P* < 0.01 was considered to be statistically significant.

## Results

### Specimen observation and statistical analysis

Normal group: the surface of articular cartilage was brightly colored, smooth, without fissures, without joint effusion and bone redundancy formation. Model group: Articular cartilage was yellow, with rough surface, cartilage defects, erosions and ulcers, joint bone redundancy formation, and a large amount of joint cavity effusion. Autologous P-PRP group: the articular cartilage was yellowish, the surface was smoother than that of the model group, with localized fibrosis, some mild bone redundancy formation, and a small amount of joint cavity fluid formation. Allogeneic P-PRP Group: articular cartilage was yellow with rough surface, severe cartilage defects extending to deep cartilage, forming ulcers, exposing subchondral bone, severe articular bone redundancy formation, and a large amount of joint cavity effusion. (Supplementary materials)

The macroscopic rating of articular cartilage in the autologous PRP group was lower than that in the model group (*P* = 0.011), and its joint effusion also improved compared with that in the model group (*P* = 0.004), and the difference between it and the normal group was not statistically significant (*P* > 0.05). In the allogeneic PRP group, the macroscopic ratings of articular cartilage, bone flab and joint effusion formation were not significantly improved compared to the model group (*P* = 0.659, *P* = 0.267, *P* = 0.553). Otherwise, the macroscopic ratings of articular cartilage, bone redundancy and joint effusion in the autologous P-PRP group were better than those in the allogeneic P-PRP group (all *P* values <0.05).

In terms of macroscopic ratings of bone redundancy, the differences between the autologous P-PRP group and the allogeneic P-PRP group were not statistically significant compared with the model group (both P values were 0.267), but the formation of bone redundancy in the autologous P-PRP group was better than that in the allogeneic P-PRP group (*P* = 0.026) (Table 1).

**Table 1:** Efficacy outcomes.

	Control	OA	Autologous PRP	Allogeneic PRP	*P*-value	Control vs. OA Adjusted *P*-value	Control vs. autologous PRP Adjusted *P*-value	Control vs. allogeneic PRP Adjusted *P*-value	OA vs. autologous PRP Adjusted *P*-value	OA vs. allogeneic PRP Adjusted *P*-value	Autologous PRP vs. allogeneic PRP Adjusted *P*-value
Macroscopic rating											
Articular cartilage	0(0-0)	2(2–2.75)	1(1-1)	2.5(2–3)	**<0** **.** **001**	**<0**.**001**	0.122	**<0**.**001**	**0**.**011**	0.659	**0**.**003**
Articular osteophyte formation	0(0-0)	2(1.25–2)	1(1–1.75)	2.5(2–3)	**<0**.**001**	**<0**.**001**	**0**.**015**	**<0**.**001**	0.267	0.267	**0**.**026**
Joint effusion formation	0(0-0)	2(2-2)	0(0–1)	2(2–3)	**<0**.**001**	**<0**.**001**	0.445	**<0**.**001**	**0**.**004**	0.553	**0**.**001**
Modified Mankin's scores	0(0-0)	11.5(10.25–12)	2.5(1–4.75)	9.5(8.25–10)	**<0**.**001**	**<0**.**001**	0.106	**<0**.**001**	**0**.**045**	0.206	**0**.**001**
Average absorbance value											
Bone morphogenetic protein 2	19.97(7.16)	12.09(4.25)	70.43(8.52)	33.58(16.70)	**<0**.**001**	0.815	**<0**.**001**	0.078	**<0**.**001**	**0**.**002**	**<0**.**001**
Sox9	73.53(20.25)	30.27(13.00)	60.94(8.80)	55.09(21.91)	**<0**.**001**	**<0**.**001**	0.729	0.185	**0**.**003**	**0**.**02**	1

*Data were expressed as mean ± SD or median (interquartile range [IQR]). Adjusted P-value was applied when pairwise comparisons in the Kruskal–Wallis test were carried out by the Bonferroni method. Bold font indicates statistically signifificant difference of P < 0.05.*

### Histological scoring and statistical analysis of HE staining

**Figure 1:**
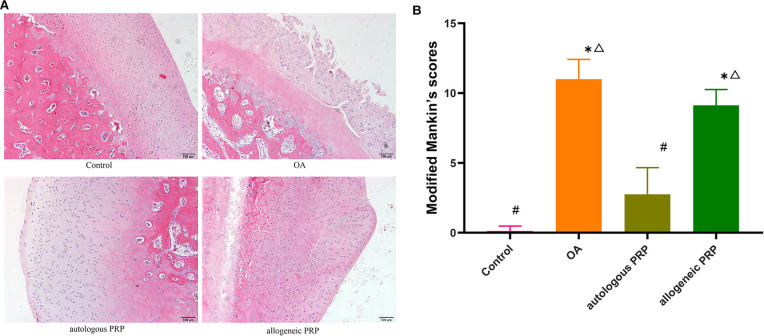
Histological images and analysis of articular facet(femur) cartilage. (**A**) H&Estained sections of articular artilage. Scale bar 100 μm. (**B**) Cartilage destruction was assessed using the Mankin scoring system. All groups (*n*=8). Groups were expressed as *p<0.01 if significantly different compared control, ^#^*p*<0.05 compared to OA, and ?*P*<0.05 compared to autologous PRP. H&E, hematoxylin and eosin; OA, osteoarthritis; PRP, platelet-rich plasma.

Normal group: most of the cartilage surface were smooth, some of the surface were irregular, chondrocytes were neatly arranged, cell size was uniform, cell matrix staining was uniform, and tide line was intact. Model group: cartilage surface was irregular, fissures appeared, cell arrangement structure was not regular, compared with the normal group, the chondrocyte layer was obviously thinner, the number of cells was reduced, local chondrocytes gathered into clusters, and the tide line was destroyed. Autologous P-PRP group: the cartilage surface was smoother than the model group, no fissures or ulcers were formed, the cell arrangement was irregular, some chondrocytes were solidified, the number of lipid degenerated cells increased, some of them appeared localized cell aggregation into clusters, the matrix staining was mildly reduced, and some tide lines were disordered. In the allogeneic P-PRP group: chondrocyte surface was rough, fissures appeared, some reached the calcified zone, chondrocyte layer was thinned, irregular arrangement, local chondrocyte aggregation into clusters, even the number of cells was too small, matrix staining was moderately reduced, and tide line integrity was disrupted (Figure 1).

As seen from the total modified Mankin's score: the modified Mankin's score in the autologous P-PRP group was reduced compared with the model group (*P* = 0.045), and there was no significant difference with the normal group (*P* = 0.106). In contrast, there was no significant change in the modified Mankin's score in the allogeneic P-PRP group compared with the model group (*P* = 0.206) (Table 1).

### Immunohistochemical analysis and statistical analysis

#### BMP-2 immunohistochemistry

**Figure 2:**
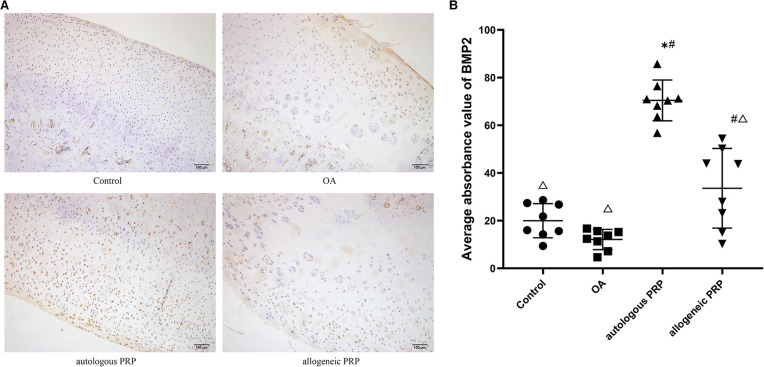
(**AB**) Immunohistochemical analysis of BMP-2 in joint fluid. Scale bar 100 µm. All groups (*n*=8). Significant differences, if any, were indicated as *p<0.05 compared control, ^#^*p*<0.05 compared to OA, and ?*P*<0.05 compared to autologous PRP.BMP-2, bone morphogeneic protein; OA, osteoarthritis; PRP, platelet-rich plasma.

Chondrocytes with positive immunohistochemical expression of BMP-2 under light microscopy were chondrocytes with brown granules attached to the nucleus, mass or membrane. Normal group: chondrocytes were evenly distributed, and some chondrocytes with light cytoplasmic staining were seen in the transition layer, and no obvious positive cells were seen in the superficial layer, radiolucent layer, and calcified layer; model group: surface was rough, cartilage stratification was not obvious, cells were disordered, local cells were clustered, and only a small amount of very light cytoplasmic staining was seen in the transition layer; autologous P-PRP group: surface was regular, chondrocytes were arranged The expression of the superficial layer and the calcified layer was relatively weak; the allogeneic P-PRP group: the surface was irregular, the local cells were clustered, the superficial layer and the transitional layer showed a large number of chondrocytes with brownish particles in the cytoplasm, the radiolucent layer showed a small amount of lightly stained cytoplasm, and the calcified layer had no expression. Immunohistochemical staining of BMP-2 was performed for each group (Figure 2).

The mean optical density values of BMP-2 in each group were calculated according to the image analysis system, and the results showed that the autologous P-PRP group had the highest expression (mean; standard deviation: 70.43; 8.52) and the model group had the lowest BMP-2 expression (mean; standard deviation: 12.09; 4.25) in each group. The number of positive cells and expression intensity in the autologous P-PRP group were significantly higher than those in the normal and model groups (all *P* values <0.01); the expression in the allogeneic P-PRP group was also higher than that in the model group (*P* = 0.002), but lower than that in the autologous P-PRP group (*P* < 0.01) (Table 1).

#### Sox9 immunohistochemistry

**Figure 3:**
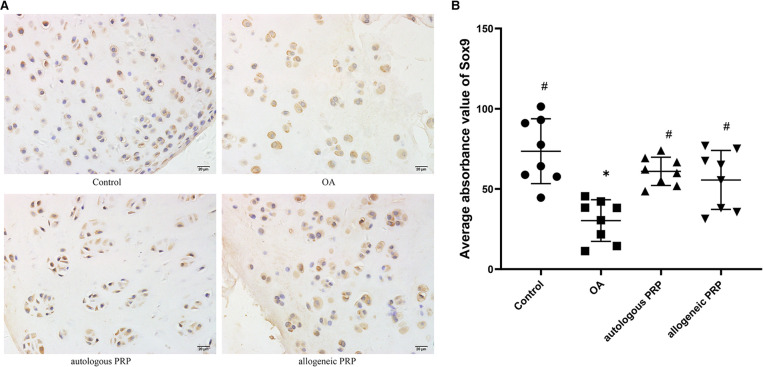
(**AB**) Immunohistochemical analysis of SOX-9 in joint fluid. Scale bar 20 µm. All groups (*n*=8). Significant differences, if any, were indicated as *p<0.05 compared control, ^#^*p*<0.05 compared to OA, and ?*P*<0.05 compared to autologous PRP. OA, osteoarthritis; PRP, platelet-rich plasma.

Positive immunohistochemical expression of Sox9 in chondrocytes under light microscopy as chondrocytes with brown granules attached to the nucleus, mass or membrane. In the normal group: chondrocytes were neatly arranged, and a large number of brown granules in the cytoplasm were visible in the whole layer of cartilage; in the model group: the surface was rough, the cell stratification was not clear, and a large number of cells gathered in clusters, and light brown coloring was seen in the cytoplasm of chondrocytes; in the autologous P-PRP group: a large number of brown granules in the cytoplasm were attached in the superficial layer, transition layer, and radiolucent zone, while the cytoplasm of cells in the calcified zone was mostly attached with light brown granules; in the allogeneic P In the PRP group, chondrocytes were disordered, with a large number of cell clusters formed and light brown granules attached to the cytoplasm of chondrocytes in the whole layer. Sox9 immunohistochemical staining of each group (Figure 3).

According to the image analysis system to calculate the mean optical density value of Sox9 in each group, the results showed that the normal group had the highest expression of Sox9 (mean; standard deviation: 73.53; 20.25), the model group had the lowest expression (mean; standard deviation: 30.27; 13), and the expression of autologous P-PRP group and allogeneic P-PRP group were higher than than the model group expression, and the difference was statistically significant (*P* = 0.003; *P* = 0.02); the differences were not statistically significant (*P* > 0.05) in the three groups of normal, autologous P-PRP and allogeneic P-PRP groups, respectively, when compared two by two (Table 1).

## Discussion

This study confirmed that cartilage repair, bone flab and joint fluid were improved in the autologous P-PRP group compared with the model group, while there wasn't significant improvement in the allogeneic P-PRP group. Immunohistochemical analysis showed that the autologous P-PRP group and allogeneic P-PRP group showed improvement compared with the model group, but the improvement was more obvious in the autologous P-PRP group. And the difference between autologous P-PRP group and allogeneic P-PRP group was statistically significant, which was consistent with the observation of large specimens, indicating that both homologous autologous and allogeneic P-PRP could improve the proliferation ability of chondrocytes in KOA and promote chondrocyte repair to some extent, but the therapeutic effect of autologous P-PRP was more obvious.

The formation and exacerbation of OA is closely related to the ongoing damage of articular cartilage. Cartilage damage leads to inflammation, and inflammatory components play an important role in the development and progression of OA. Studies have shown that the intra-articular environment of catabolism and inflammation present in osteoarthritis can be affected by PRP [18]. Intra-articular injection of PRP is a modern therapeutic technique for KOA, and it has been shown that activated P-PRP can effectively promote the proliferative repair capacity of chondrocytes and modulate the intra-articular inflammatory response. P-PRP contains a large number of growth factors with anti-catabolic and anti-inflammatory effects, such as platelet growth factor that stimulates hyaluronic acid synthesis by synovial fibroblasts; leukocytes that inhibit NF-*κ*B gene activation interleukin-1 receptor antagonist, thus participating in apoptosis and inflammatory processes; transforming growth factor *β*, which inhibits cartilage degradation and enhances the expression of tissue metalloproteinase inhibitors. In addition, there is insulin-like growth factor-1, which regulates the metabolism of chondrocytes and subchondral bone, maintains homeostasis between synthesis and degradation of proteoglycans, and stimulates chondrocyte proliferation [19–21]. In vitro experimental studies on knee chondrocytes showed that platelet-rich plasma intervention effectively inhibited Wnt/*β*-catenin signaling and significantly enhanced the proliferation of chondrocytes [22]. In an *in vivo* experiment in a KOA mouse model, PRP injection in the joint cavity was found to effectively reduce the catabolic activity of chondrocytes and slow down the rate of chondrocyte apoptosis [23]. In addition, researchers also that platelet-rich plasma can reduce pain and synovial thickness by regulating macrophage subtypes through experiments in a mouse OA model [24]; a number of clinical studies have also continued to report the therapeutic value of PRP for KOA [8–11]^.^

Currently, most of the PRP used for clinical treatment is prepared using autologous blood. However, some investigators have found that allogeneic P-PRP for the repair of bone defects can also achieve good efficacy [25–27]. In a randomized controlled clinical trial, allogeneic PRP was used in combination with standard care in chronic refractory wounds, and the results showed a significant reduction in inflammatory exudation and a significant reduction in healing time in wounds treated with allogeneic PRP [28]. Jo et al. also used allogeneic PRP in arthroscopic injury repair of rotator cuff injuries, and the results showed that allogeneic PRP not only had the same efficacy to autologous PRP, but also did not cause any local or general complications [29]. In a study of allogeneic PRP repair of Achilles tendon injury in rats, it was reported that a single injection of allogeneic P-PRP was effective in promoting collagen deposition in Achilles tendon protofibrils while enhancing the tensile strength of the Achilles tendon [30]. In an animal experiment on osteoarthritis of the guinea pig knee, Kanwat et al. injected allogeneic PRP into the knee cavity of the affected limb and showed that synovitis was significantly improved and knee synovial fluid COMP levels were significantly reduced in a short-term analysis, but the beneficial effects of PRP observed in the short-term were not reproduced in long-term observations [31]. Thus, the effect of allogeneic PRP on KOA articular cartilage remains ambiguous.

Nowadays several cytokines have been shown to be involved in chondrocyte anabolism [32, 33]. BMP-2 can effectively induce chondrogenesis in human mesenchymal stem cells and is considered as a protective factor for articular cartilage [34, 35]; Sox9 is a core transcription factor that regulates chondrogenesis and development and mainly regulates chondrocyte differentiation, so BMP-2 and Sox9 are closely related to chondrocyte regeneration [36]. The immunohistochemical results of this study showed that the expression of BMP-2 and Sox9 in the model group was the lowest, considering the reason for the poor proliferation and repair ability of chondrocytes after cartilage injury in the KOA model; the expression of BMP-2 and Sox9 increased after intra-articular injection of autologous P-PRP, and there was a large amount of expression in both the cartilage transition layer and the radiolucent layer, suggesting that P-PRP may upregulate BMP-2 and Sox9 through the expression to improve the proliferation of chondrocytes and promote cartilage injury repair; whereas, although the expression of BMP-2 and Sox9 was increased after allogeneic P-PRP treatment, BMP-2 was mainly expressed in the chondrocyte transitional layer, and there was a difference in its expression compared with the autologous P-PRP group. The results of bulk specimens and histological scoring also showed no significant improvement in the allogeneic P-PRP group compared to the model group. A previous study found that repeated injections of BMP-2 into the normal murine joint cavity resulted in bone redundancy formation on the murine articular surface and strong expression of BMP-2 in the bone redundancy of late-stage KOA [37]. Therefore, BMP-2 expression in OA chondrocytes plays a different significance, the expression of this factor is low in healthy cartilage and increased in cartilage injury, OA cartilage, firstly, it is considered that BMP-2 in upregulated as a repair response after injury stimulation, but the possibility that BMP-2 stimulates further development of OA injury cannot be excluded; therefore, it is speculated that the expression of BMP-2 in the allogeneic P-PRP group in this study is concentrated in the transitional layer, which cannot effectively improve the repair of injured cartilage and may also induce the formation of bone redundancy on the articular surface, but whether this inference is valid needs to be confirmed by further studies.

## Conclusions

Intra-articular injection of autologous P-PRP can effectively activate the expression of BMP-2 and Sox9 in chondrocytes to promote chondrocyte proliferation and cartilage repair in order to reduce the production of bone flab and joint fluid for the treatment of KOA. However, the efficacy of allogeneic P-PRP on KOA in rabbits is not optimistic, and the underlying causes are yet to be explored in high-quality related experiments.

### Limitations

The main shortcomings of this experiment are as follows. Firstly, the number of samples included in this experiment is small. Second, due to the limited study conditions, the experiment failed to detect platelet antibodies in tissue specimens, which led to the failure to clarify the underlying causes of the differences in the efficacy of homologous autologous and allogeneic P-PRP for the treatment of KOA. Third, this study only took the specimens for observation 2 weeks after the last injection, and no long-term experimental results analysis was performed, which will be further improved and perfected in the follow-up study.

## Data Availability

The original contributions presented in the study are included in the article/Suplementary Material, further inquiries can be directed to the corresponding author/s.
